# FOK l Vitamin D Receptor Gene Polymorphism and Risk of Dental Caries: A Case-Control Study

**DOI:** 10.1155/2022/6601566

**Published:** 2022-08-05

**Authors:** Nireeksha Nireeksha, Mithra N. Hegde, Shilpa S. Shetty, Suchetha N. Kumari

**Affiliations:** ^1^AB Shetty Memorial Institute of Dental Sciences, Nitte (Deemed to Be) University, Deralakatte, Mangalore 575018, India; ^2^Department of Conservative Dentistry and Endodontics, AB Shetty Memorial Institute of Dental Sciences, Nitte (Deemed to Be) University, Deralakatte, Mangalore 575018, India; ^3^Central Research Laboratory, KS Hegde Medical Academy, Nitte (Deemed to Be) University, 28 Deralakatte, Mangalore 575018, India; ^4^Department of Biochemistry, KS Hegde Medical Academy, Nitte (Deemed to Be) University, Deralakatte, Mangalore 575018, India

## Abstract

The prevalence of dental caries in individuals who practice good oral hygiene increasingly indicates that other etiological factors, such as genetic factors, may be responsible for occurrence of caries, and its prevalence in younger individuals, such as adolescents, is an early manifestation of their genetic makeup. Therefore, there is a need to investigate the correlation of various genetic factors with the occurrence of dental caries in populations. Thus, this study assessed the relationship between single nucleotide polymorphism (rs2228570) in the vitamin D receptor gene and dental caries susceptibility. After obtaining ethical approval (NU/CEC/2020/0339), 377 adults, aged 18–40 years, were included in this study. Among the participants consenting to participate, salivary samples were collected, and an oral examination was conducted using the World Health Care Oral Health Survey Format 2013. The DMFT and PUFA index scores were recorded along with basic demographic details. The subjects were categorized as caries-free (controls, DMFT = 0) and caries-active (cases). The case group was further divided into the high-risk group (DMFT ≤ 10), moderate-risk group (DMFT = 4–9), and low-risk group (DMFT = 1–3). Saliva samples were used for vitamin D level analysis and DNA isolation. Polymerase chain reaction-based restriction fragment length polymorphism analysis using Fok1 digestion was performed on the isolated DNA. Salivary vitamin D levels were markedly higher in the caries-free group than in the caries-active group (*p* < 0.001). The T allele of rs2228570 was significantly associated with having active caries, while the C allele was associated with being caries-free. Individuals with the rs2228570 TC genotype had 2.814-fold increased likelihood, and individuals with the TT genotype had 3.116- fold increased likelihood of being caries-active. This finding is important in terms of patient counselling, as well as possibly in terms of prevention and treatment of caries.

## 1. Introduction

The World Health Organization (WHO), in the Global Burden of Diseases list of 2017, indicated that untreated dental caries in permanent teeth is one of the most common health conditions globally. Thus, this is an area in healthcare that requires increased attention, particularly in low-income and middle-income countries [1, 2]. The key strategies to tackle oral health problems by the WHO include population-based strategies to decrease intake of caries-inducing food items, analyzing the impact of the problem, and surveying factors related to prevention of dental caries [3]. The pathophysiology and reason for the development and progression of caries have been well explained over the years in various contexts. The multifactorial processes involved in dental caries make it a dietary-microbial disease with various underlying factors, including psychological, behavioral, social, and genetic factors [4, 5].

Untreated dental caries in permanent teeth is responsible for various outcomes, such as severe pain, malocclusion, abscesses, and tooth loss [6]. However, individuals with similar exposure to dietary food, cariogenic bacteria, and other environmental risk factors vary in their susceptibility to caries [6]. The varying susceptibility to caries development even in individuals exposed to fluoride [7] has indicated the need for considering that genetic factors may contribute to dental caries occurrence and prevalence. Understanding host susceptibility, i.e., the contribution of various genetic factors, increases the ability of clinicians to explain the role of genetic and inherited risk factors in a disease [8].

**Figure 1:**
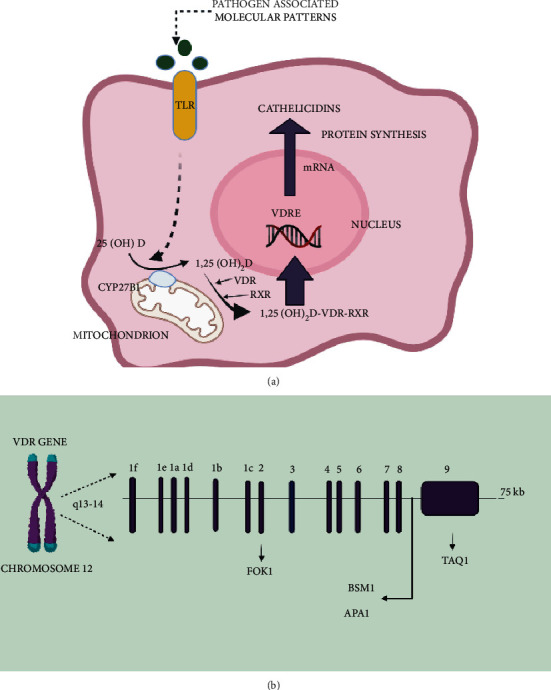
(a) Vitamin D cathelicidin pathway (TLR2 vitamin D LL-37). (b) Locations of various single nucleotide polymorphisms in *VDR* (created by BioRender).

Vitamin D, through its two major roles, is identified as a risk factor for dental caries occurrence and prevalence [9]. It plays a key role in different avenues in the oral cavity, including (a) tooth mineralization, (b) antibacterial effects through the release of antimicrobial peptides, and (c) effects on ameloblasts and odontoblasts, i.e., formation of enamel and dentin via signaling pathways. Thus, vitamin D regulates the immune response, in addition to modulating calcium metabolism [10]. As a ligand-dependent transcription regulator, the vitamin D receptor mediates the cellular function of 1,25 (OH)_2_ D_3_ distributed in the cytoplasm. It binds to the vitamin D receptor element to perform various functions [11], which include bone mineralization, improving homeostasis, increasing absorption of calcium and phosphate, decreasing levels of parathyroid hormone, and release and differentiation of immune cells (Figure 1).

The ligand-activated transcription factor defines the biological activity of 1,25 (OH)_2_ D_3_. The major steps involved in gene transcription by the vitamin D receptor are ligand binding and heterodimerization with the retinoid X receptor (RXR), which then binds to the vitamin D response element and then recruits other nuclear proteins into the transcriptional preinitiation complex. This shows how variation in *VDR* could affect numerous processes [12]. Hence, we considered it appropriate to evaluate the association of polymorphisms in *VDR* with biochemical levels of vitamin D in saliva and with dental caries.

**Figure 2:**
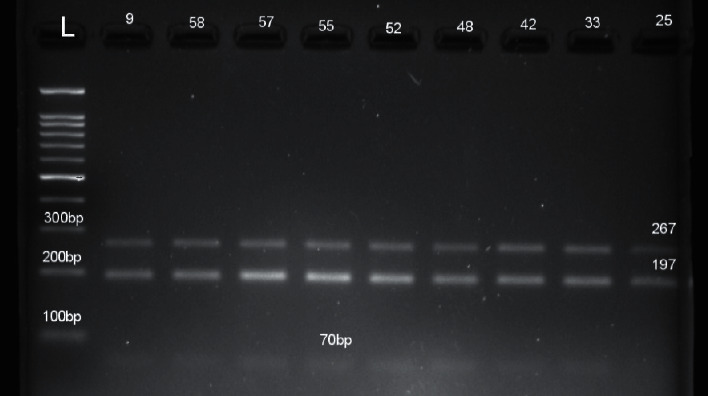
Representing RFLP for FOK1 gene polymorphism.

Genetic variations which appears at least 1% in the population is called polymorphism. Some of these variations create or remove restriction enzyme sites in DNA [13]. Thus, digestion of DNA containing these variants with restriction enzymes yields different lengths and fragments of DNA, and thus, these polymorphisms are termed restriction fragment length polymorphism (RFLPs). Single nucleotide polymorphisms in *VDR* affect Taq1, Apa1, and Bsm1 sites in exons 8 and 9 (Figure 2), whereas the Fok1 site is affected by polymorphism (rs2228570) located in exon 2 [14]. Here, we investigated the relationship between this Fok1 RFLP in *VDR* locus and dental caries susceptibility, using a case-control study.

## 2. Methods

### 2.1. Study Subjects

This research was approved by the university's ethical committee (NU/CEC/2020/0339). This study included 377 adults, aged 18–40 years. The sample size was calculated to ensure 90% power with a 95% confidence interval, using the formula *n* = 4*pq*/*d*^2^ and considering the prevalence of dental caries [15, 16]. Individuals visiting the outpatient department for regular dental checkups and treatments were included based on inclusion and exclusion criteria. We included healthy individuals free of associated systemic conditions and who were not taking any nutritional supplementation. We excluded lactating or menopausal women, as well as smokers.

Patients were provided with an information sheet that described the details of the study and the usage of salivary samples. After an oral examination, salivary samples were collected from participants who provided consent. Furthermore, the subjects were divided into cases and controls based on caries experience. The subjects were categorized into controls if their decayed, missing, and filled teeth (DMFT) score was 0, whereas the case group was subdivided into the high-risk group (DMFT ≥ 10), moderate-risk group (DMFT = 4–9), and low-risk group (DMFT = 1–3).

### 2.2. Questionnaire

Baseline data were obtained, including detailed demographic data (age, sex, height, and weight) and dietary habits (nonvegetarian/vegetarian), and details of oral hygiene practices.

### 2.3. Dental Examination

Teeth that were decayed, missing, or filled due to caries were assessed and recorded according to the Oral Health Survey 2013 criteria (Annexure I). The DMFT and PUFA indexes were monitored [17, 18]. The PUFA index measures the oral condition associated with untreated caries (visible pulpal involvement (P), ulceration caused by dislocated tooth fragments (U), fistula (F), and abscess (A)). This index indicates the extent of the severity of dental caries, rather than merely focusing on the presence or absence of caries.

The patients were asked to sit on a dental chair, the food debris on the tooth surfaces was removed with sterile cotton, and the teeth were dried to observe white spot lesions and caries. No radiographs were taken.

### 2.4. Saliva Sample Collection

Unstimulated saliva samples were collected using the Navazesh protocol [19]. Participants were asked to abstain from smoking, brushing of teeth, use of mouthwash, and eating or drinking for 2 h prior to the sample collection. The samples were collected between 10.00 am and 11.00 am. During the sample collection, participants were seated on a normal chair rather than a dental chair to maintain a stress-free environment. Once unstimulated saliva had pooled in the floor of the mouth, 5 ml was collected in the Tarson's saliva collection tube. The saliva samples were centrifuged, and the supernatant obtained was stored at −4°C for subsequent analysis.

### 2.5. Biochemical Analysis of Vitamin D

Salivary vitamin D was analyzed using a commercially available vitamin D enzyme-linked immunosorbent analysis kit (Epitope Diagnostics, Inc., San Diego, CA, USA).

### 2.6. Salivary DNA Isolation

Salivary DNA was isolated using a commercially available kit (Salivary DNA Isolation Kit, Norgen Biotek, Thorold, ON, Canada) as per the manufacturer's instructions. The DNA concentration in each sample was analyzed using a biospectrophotometer and was stored at −20°C until further analysis.

### 2.7. Single Nucleotide Polymorphism Selection and Genotyping

The single nucleotide polymorphism (SNP) in *VDR* was selected based on previous publications and was obtained from https://www.ncbi.nlm.nih.gov. DNA was amplified by polymerase chain reaction (PCR) and digested with the restriction enzyme to evaluate the RFLP. The primer pair for amplifying DNA flanking of Fok1 SNP (rs2228570) was as follows: F: 5′-AGC TGG CCCTGG CAC TGACTC GCT CT-3′ and R: 5′- ATGGAA ACA CCT TGC TTC TTC TCC CTC-3′. The 25-*μ*l PCR mixture consisted of 10 mM TrisHCl, 200 *μ*M dNTPs, 20 pmol of each primer, 1.5 mM MgCl_2_, 0.5 U Taq polymerase (F enzyme), and 50–100 ng of DNA as a template. The cycling profile was as follows: 5 min at 94°C, followed by 35 cycles of 95°C for 60 s, 68°C for 60 s, and 72°C for 2 min, followed by 72°C for 7 min as the final extension step. Ten microliters of the PCR-amplified product was digested with restriction enzymes at 37°C overnight. The digested product was subsequently visualized on 3% agarose gel stained with ethidium bromide.

### 2.8. Statistical Analysis

Collected data are summarized using the frequency (percentage) or mean and standard deviation. Comparisons were made using the chi-square test, analysis of variance, or the *t*-test. Logistic regression analysis was performed to obtain the odds ratio for association of genotypes with cases. Receiver operating characteristic (ROC) curve analysis was performed to obtain the optimum cutoff vitamin D level, with the corresponding sensitivity and specificity. SPSS (version 23; IBM SPSS Corp, Armonk, NY, USA) software was used to perform statistical comparisons.

## 3. Results

The study examined 239 cases (caries-active) and 138 controls (caries-free) individuals, of which, 239 were females and 138 males, and age and sex were matched (Tables 1 and 2).

### 3.1. Biochemical Characteristics of the Study Population

Among the individuals in the caries-active group, the mean salivary vitamin D level was 20.85 pg/ml as compared to 28.56 pg/ml in the caries-free group (*p* < 0.001). However, the mean salivary vitamin D levels were not statistically different among the subgroups in the caries-active subgroups (Table 3). We performed ROC analysis to find a vitamin D value that can distinguish the caries-active group from the caries-free group. We found that the optimal cutoff value was 28.33 pg/ml, with a sensitivity of 71% and specificity of 57% and an area under the curve of 0.694.

### 3.2. Genetic Polymorphism and Its Association with Caries Prevalence

Genotype frequencies for rs2228570 were not in agreement with the Hardy–Weinberg equilibrium among the caries-free controls (*p*=000, chi-square 17.46). Among the caries-active individuals, the genotypes were distributed as follows: CC (63.2%), TC (12.1%), and TT (24.6%). In the caries-free control group, the genotypes were distributed as follows: CC (83.8%), TC (5.7%), and genotype TT (10.5%) (Table 4). The rs2228570 genotypes were significantly different between the case and control groups according to the chi-square test (Table 5).

The odds ratios were calculated by logistic regression analysis to prove the significance of association of the different rs2228570 genotypes with the caries status in the case and control groups. Genotype CC was used as the reference. Individuals with genotype TC had 2.814-fold increased likelihood of having active caries, while those with genotype TT had 3.116-fold increased likelihood of having active caries (Table 6).

Genotype models showed no significant association with salivary vitamin D levels, although they did show significant association with the caries status (Table 7).

Additionally, there was a significant association of the T allele with the caries-active status and of the C allele with the caries-free status.

## 4. Discussion

Genetic factors may contribute markedly to the multifactorial nature of dental caries, but the role of genetic factors in caries development has not been well studied [20]. Vitamin D plays a major role in calcium homeostasis that is regulated by vitamin D and also plays a major role in the immune response and has anti-inflammatory activity [21]. Vitamin D receptor gene polymorphism varies among different ethnicities. These polymorphisms have been associated with bone phenotype, hormonal homeostasis, diet, and exposure to the sun [22–25]. In this study, salivary vitamin D levels were markedly higher in the caries-free group than in the caries-active group (*p* < 0.001). We found that the T allele of rs2228570 was significantly associated with the active caries status, while the C allele was associated with the caries-free status. The likelihood of having active caries was increased 2.814-fold and 3.116-fold in individuals with the rs2228570 TC and TT genotypes, respectively. Individuals in both the caries-free and caries-active groups showed similar practices, such as tooth-brushing once a day, nonsignificant intake of food between meals, and nonsignificant intake of sugar and sticky foods, based on patients' self-reporting. Thus, the effects of these factors did not contribute markedly to the outcomes. In the present study, salivary vitamin D levels were significantly higher in the caries-free group than in the caries-active group. This can be attributed to the production of protective peptides LL-37/cathelicidins, due to activation of the TLR2-vitamin D-LL-37 pathway, where production of these peptides occur due to binding of 1,25 (OH)_2_ D to the vitamin D receptor. This increases the antimicrobial capacity of immune cells, such as neutrophils, and decreases the chances of new carious lesions developing. Thus, the increased levels of salivary vitamin D indicate the efficiency of antimicrobial functions [26]. Vitamin D also stabilizes the demineralization and disintegration of tooth surfaces, maintaining adequate surface proteins by upregulating expression of various proteins, such as enamelins, dentin sialoproteins, amelogenins, enamelins, and dentin phosphoproteins. In the present study, the salivary levels of vitamin D can be attributed to normal to average exposure to sun and through various food resources [27]. These results were in agreement with those of Gyll et al. who concluded that insufficient salivary vitamin D correlates with significant carious lesions [28]. Kim et al. also found that vitamin D levels lower that 50 nmol/L posed a high risk of caries in children with permanent dentition [29]. *VDR* contains various polymorphisms. We found a clear association of the Fok1 RFLP in *VDR* TC and TT genotypes with active caries. This finding can help identify the role of host susceptibility, genetically driven immune deficiency, and inflammatory changes in the incidence of caries in individuals [30]. The presence of variation at the start site of the gene due to Fok1 RFLP may result in production of diverse sizes of vitamin D protein. In a similar study conducted by Yu et al. in the Chinese population, this SNP showed a significant association with dental caries in permanent dentition. They found an increased frequency of the CT and CC genotypes in the caries group, whereas the TT genotype and T allele frequencies were significantly reduced in the caries group, as compared with the caries-free controls [31]. In a meta-analysis that studied the correlation of all SNPs in *VDR* with dental caries, only Fok1 SNP showed a significant correlation with dental caries, which can be attributed to the location and cotranscription factors [14]. Das et al. investigated the frequency of the Fok1 and Taq1 RFLPs in *VDR* in healthy Indian individuals and their association with 25-OH-vitamin D levels. They found a significant association of Taq1 RFLP but not Fok1 RFLP with 25-OH-vitamin D levels [32]. Barbosa et al. conducted a study in permanent dentition to evaluate the association of the Fok1 and Bg11 RFLPs in *VDR* with dental caries and concluded that there was no evidence of a statistically significant association [20]. In this study, dental caries status was evaluated retrospectively; i.e., the “missing” and “filled” teeth were included with the decayed teeth when patients presented a history of extraction due to caries and filling due to dental caries. The study was limited by its sample size. Further studies should be performed in larger groups. Additionally, intervention studies with vitamin D supplementation should be performed in the future. In the future, prospective studies should evaluate the association of these polymorphisms with antimicrobial peptides and analyze the TLR2-vitamin D pathway, to establish whether these genotypes have functional effects on the biochemical levels of vitamin D and cathelicidins. The strength of the study was the association of genotypes with functional biochemical levels of vitamin D levels. Additionally, estimation of vitamin D and isolation of DNA from saliva is noninvasive and may be more convenient for individuals reporting for dental checkup, who are not willing to undergo venous blood withdrawal for analysis. The concept of investigating genetic makeup or SNPs in routine visits through easy diagnostic tests may help diversify treatment protocols and provide a preliminary understanding of caries susceptibility in even very young individuals.

## 5. Conclusion

In the present study, we determined that the TC and CC genotypes of Fok1 RFLP (rs2228570) in *VDR* predispose individuals to fall into the caries-active group. This finding emphasizes the focus on genetic polymorphism, whereby clinicians can explain to patients their likelihood of caries development and promote strategies to improve oral hygiene habits with the aim of decreasing the prevalence of dental caries. Host susceptibility can be clearly explained to the patient. Additionally, if SNP can be shown to have affected the functional levels of vitamin D and antimicrobial peptides, supplementation of these factors may possibly address the effect of genetic variations. This study emphasizes that while dental caries are multifactorial, host susceptibility in the prevalence of caries is an important consideration.

## Figures and Tables

**Table 1:** Age group and sex distribution of participants.

		Group					
		Controls		Cases		Total	
		Count	%	Count	%	Count	%
Sex	F	67	63.8	172	63.2	239	63.4
	M	38	36.2	100	36.8	138	36.6
Age group (years)	18–25	70	66.7	190	69.9	260	69.0
	26–35	20	19.0	47	17.3	67	17.8
	36–40	15	14.3	35	12.9	50	13.3

**Table 2:** The significance of the age group and gender match in the study.

Group	Chi square value	d.f	*p*
Sex	0.011	1	0.917
Age group	0.360	2	0.835

**Table 3:** Comparison of the mean vitamin D levels in the caries-free and caries-active groups.

Vitamin D	*N*	Mean	Std. deviation	95% confidence interval for mean		^*∗*^ *p* value	
				Lower bound	Upper bound		
Controls	105	28.56	10.42	26.54	30.58	0.001	HS
Cases	272	20.85	11.20	19.51	22.18		
Total	377	23.00	11.51	21.83	24.16		

A *p* value ≤0.05 was considered statistically significant. The symbol ^*∗*^ indicates Student's *t*-test.

**Table 4:** Association of genotypes rs2228570 based on Fok1 RFLP evaluation in the caries-active cases and caries-free controls.

Fok1 (rs2228570) genotypes	Cases		Controls	
	Count	Column *N*%	Count	Column *N*%
CC	172	63.2	88	83.8
TC	33	12.1	6	5.7
TT	67	24.6	11	10.5
Total	272	100.0	105	100.0

**Table 5:** Significance of association of rs2228570 with the caries status.

Genotype	Chi-square test *p* value	
FokI	0.001	HS

**Table 6:** Logistic regression analysis-based odds ratios of the significance of genotypes of rs2228570 with the caries status.

		Sig.	Exp (B)	95% CI for Exp (B)	
				Lower	Upper
Step 1^a^	rs2228570 CC genotype	0.001			
	rs2228570 TC genotype	0.025	2.814	1.136	6.970
	rs2228570 TT genotype	0.001	3.116	1.567	6.197
	Constant	0.000	1.955		

**Table 7:** Frequency of genotype models in association with vitamin D levels in the cases and controls.

		Group					
		Controls			Cases		
		Vitamin D level			Vitamin D level		
		Count	Mean	Standard deviation	Count	Mean	Standard deviation
rs2228570 genotype	CC	88	29.09	10.55	172	21.66	10.82
	TC	6	25.49	11.85	33	18.51	11.31
	TT	11	26.00	8.71	67	19.90	12.01
	ANOVA *p* value	0.498			0.244		
rs2228570 genotype	CC + TC	94	28.86	10.60	205	21.16	10.93
	TT	11	26.00	8.71	67	19.90	12.01
	*t*-test *p* value	0.392			0.427		
rs2228570 genotype	CC	88	29.09	10.55	172	21.66	10.82
	TT + TC	17	25.82	9.56	100	19.44	11.75
	*p*	0.238			0.115		

## Data Availability

The data are available from the corresponding author on request.
